# Transcriptome analysis of the key role of GAT2 gene in the hyper-accumulation of copper in the oyster *Crassostrea angulata*

**DOI:** 10.1038/srep17751

**Published:** 2015-12-09

**Authors:** Bo Shi, Zekun Huang, Xu Xiang, Miaoqin Huang, Wen-Xiong Wang, Caihuan Ke

**Affiliations:** 1 State Key Laboratory of Marine Environmental Science, Xiamen University, Xiamen 361102, PR China; 2 College of Ocean and Earth Sciences, Xiamen University, Xiamen 361102, PR China; 3 School of Life Sciences, Xiamen University, Xiamen 361102, PR China; 4 Division of Life Science, The Hong Kong University of Science and Technology, Hong Kong, PR China

## Abstract

One paradigm of oysters as the hyper-accumulators of many toxic metals is the inter-individual variation of metals, but the molecular mechanisms remain very elusive. A comprehensive analysis of the transcriptome of *Crassostrea angulata* was conducted to reveal the relationship between gene expression and differential Cu body burden in oysters. Gene ontology analysis for the differentially expressed genes showed that the neurotransmitter transporter might affect the oyster behavior, which in turn led to difference in Cu accumulation. The ATP-binding cassette transporters superfamily played an important role in the maintenance of cell Cu homeostasis, vitellogenin and apolipophorin transport, and elimination of excess Cu. Gill and mantle Cu concentrations were significantly reduced after silencing the *GABA transporter 2 (GAT2)* gene, but increased after the injection of GABA receptor antagonists, suggesting that the function of *GABA transporter 2* gene was strongly related to Cu accumulation. These findings demonstrated that GABA transporter can control the action of transmitter GABA in the nervous system, thereby affecting the Cu accumulation in the gills and mantles.

Copper (Cu) is a micronutrient essential for all living organisms, but can also be toxic when present in excess by generating hydroxyl radicals^1^. Due to human activities, an increasing amount of industrial effluents is released into rivers and coastal waters, and high levels of Cu have been reported in some aquatic ecosystems such as the Restronguet Creek in southwest Cornwall, UK, and the Jiulong River estuary, Fujian, China^2^,^3^,^4^,^5^. Oysters are one of the most important cultivated mollusk species around the world. Based on the Fishery and Aquaculture Statistics 2012 (FAO Yearbook), oyster production in China amounted to 3,949,000 t, accounting for 83% of the total world production. Due to its sedentary and filter-feeding behavior, high bioaccumulation potential, and easy collection, oysters have been extensively employed in metal contamination monitoring^6^. Oysters are considered as the hyper-accumulators of various metals, especially for Cu, zinc (Zn), and cadmium (Cd). The classic example is the ‘green-sick’ oyster (an indicative of Cu accumulation) reported in several estuaries around the world^7^,^8^. Another example is the blue colored oysters with a Cu concentration as high as 14,000 μg/g^5^,^6^.

One paradigm in marine biomonitoring is that metal concentrations in bivalves can vary greatly among different species and different individuals. Biokinetic model has been used to explore the inter- and intra-species differences in metal concentrations in oysters and other aquatic animals^9^,^10^,^11^. Luoma and Rainbow compared the biokinetics of green mussels (a relatively moderate Zn accumulator) and barnacles (a Zn hyper-accumulator) and suggested a negative relationship between the Zn body burden and the efflux rate^10^. Pan and Wang reported a 5-fold inter-individual variation in Cu body burden in the oyster *Saccostrea cucullata* with similar age, shell length and dry weight^11^. Such individual difference was not related to gender, but could be explained by the variation in efflux, with a lower efflux resulting in a very high Cu body burden. The molecular mechanism underlying such inter- and intra-species difference in Cu bioaccumulation is however unknown. Presently, there is an increasing number of transcriptome and proteome analyses in bivalves, which indicated that transcripts or networks of proteins responded to Cu and other metals^12^,^13^,^14^,^15^,^16^. These studies focused on identifying the mechanisms responsible for organisms to adapt to or alleviate the damage due to metal (Cu, Zn, and Cd) stress and accumulation in marine organisms. The transcriptome study can provide information of the potential functional genes and gene regulatory mechanisms underlying the inter- and intra-species differences in metal accumulation in marine bivalves.

The aim of this study was therefore to gain insight into the molecular mechanisms of the intra-species differences in Cu body concentrations in oyster *Crassostrea angulata* and to identify the key genes related to Cu accumulation. We performed a comprehensive analysis of the transcriptome of oysters using gills and mantle tissues. Digital gene expression (DGE) technology was applied to analyze the relationships between gene expression and differential Cu body concentrations. To analyze the differentially expressed genes (DEGs), AgriGO and KEGG functional enrichment analysis was conducted. The function clustering entries related to the behavioral change, Cu iron transport and Cu stored components were identified. According to the functional categories of these DEGs, our analysis focused on the genes involved in lipid transporter activity, Cu ion binding, neurotransmitter activity, and ATPase activity. We focused on GABA transporter 2 (GAT2), which is related to neurotransmitter activity and therefore may play an important role in Cu accumulation in oysters. In addition, we evaluated the changes in Cu body concentration in the oyster resulting from *GAT2* gene interference. GABA and GABA receptor agonists or antagonists were injected to validate the neurotransmitter-relevant candidate genes that may play important roles in the intra-species differences in Cu accumulation. To our knowledge, this is the first study that integrates transcriptome and intra-species differences in Cu body concentration in marine bivalves to provide further insights into the mechanism of Cu accumulation.

## Results

### Cu concentrations in oyster tissues used for DGE analysis

**Figure 1:**
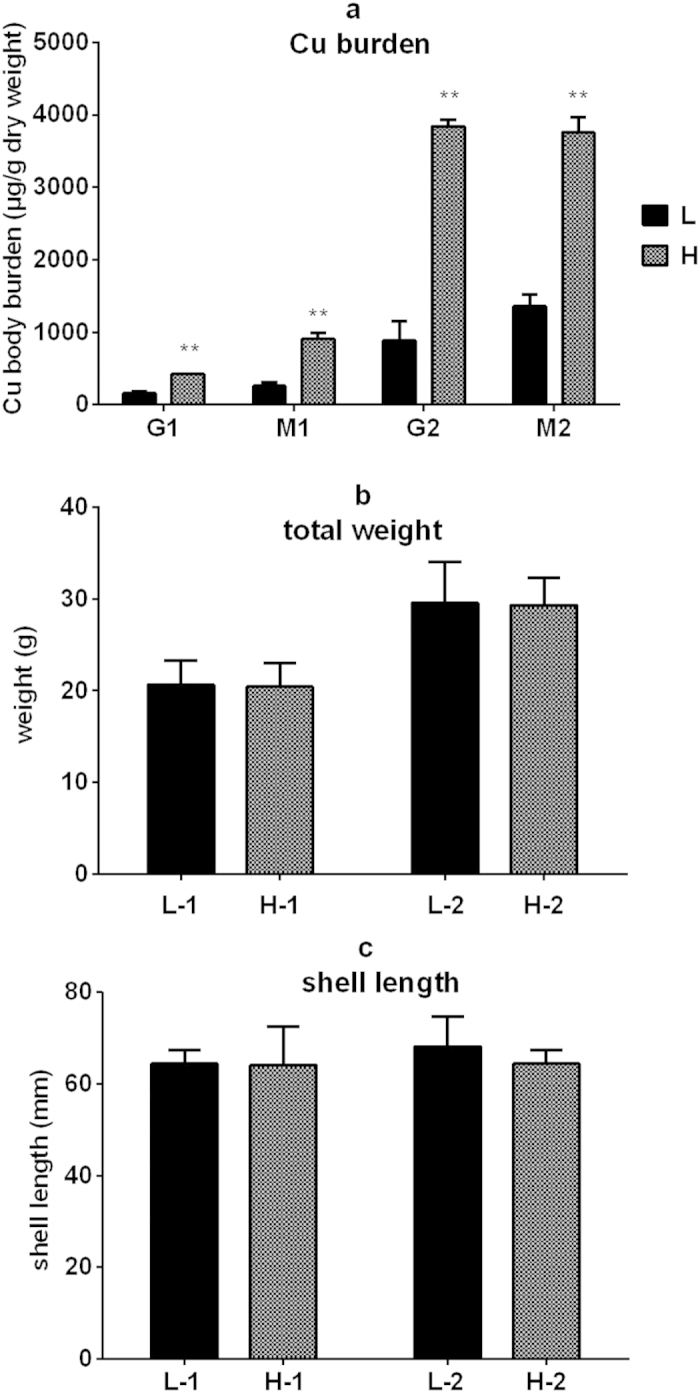
The tissue Cu concentration (**a**), total body weight (**b**) and shell length (**c**) of oysters selected for DGE. G1 and M1: gills and mantle tissues without experimental Cu exposure; G2 and M2: gills and mantle tissues after Cu exposure; L and H represent low and high Cu tissue concentration, respectively. L-1 and H-1: oysters without experimental Cu exposure; L-2 and H-2 oysters after Cu exposure. Data are mean ± s.d. (six replicates); **p <0.01 (pair-wise t test).

Individuals with the largest differences in Cu body concentration were used for differential gene expression analysis. Sixty individuals collected from an oyster farming site (as G1 and M1) were dissected and measured for Cu concentrations in the gills and mantles. Six oysters with the highest Cu concentrations in their gills and mantles were selected. Their gills were pooled and flagged as G1-H group, and mantles were pooled as M1-H. Similarly, six oysters with the lowest Cu concentrations in their gills and mantles were selected and their gills were pooled and flagged as G1-L group, and mantles were pooled as M1-L. The 60 individuals exposed to Cu (as G2 and M2) were also subjected to the same expression analysis. G2-L and M2-L groups were pooled from 6 oysters’ gills and mantles that showed the lowest Cu body concentrations. G2-H and M2-H groups were pooled from highest Cu body concentration individuals. Figure 1a shows the Cu concentrations in the gills and the mantle used for DGE analysis. Figure 1b,c show their total weights and shell lengths. No visible phenotypic differences were observed, whereas large variations in body Cu concentrations (2.7- to 4.3-fold) were detected. These groups (G1-H *vs.* G1-L; M1-H *vs.* M1-L; G2-H *vs.* G2-L; and M2-H *vs.* M2-L) showed significantly different Cu concentrations (*p* <0.01, pair-wise *t* test). Oysters subjected to a 30-day Cu exposure accumulated higher Cu concentration than the baseline Cu body concentration (e.g., Cu in G2 an M2 groups were higher than that in G1 and M1 groups, Fig. 1a). The results of the DGE analysis therefore showed that individuals within the same population had large variations in Cu concentrations.

### Sequencing and identification of differentially expressed genes (DEGs)

**Figure 2:**
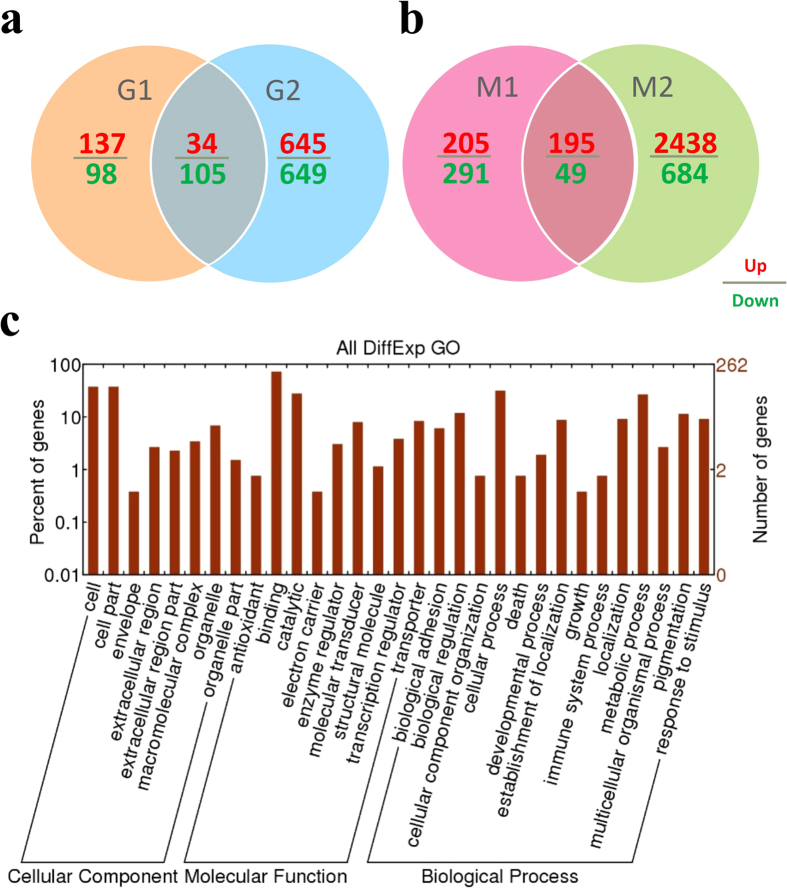
Significant differentially expressed genes (DEGs). (**a**) DEGs of oyster gills with great variation in the body burden of Cu by Venn diagram: G1-L *vs.*G1-H ∩ G2-L *vs*. G2-H. (**b**) The DEGs in high Cu accumulated mantles compared to low Cu accumulated groups: M1-L *vs.* M1-H ∩ M2-L *vs.* M2-H. A FDR of ≤0.001 and an absolute value of the log2 ratio >2 were used as the threshold to determine significant differences in gene expression. (**c**) Gene ontology analysis of all DEGs in the high-Cu and low-Cu oysters.

The Illumina Genome Analyzer was used to perform high-throughput Tag-seq analysis of the gills and mantle libraries. The major characteristics of eight libraries are summarized in the Supplementary Table S1. A total of 5.8–6.3 million reads of 50 bp in length was generated for each sample, and the number of mapped reads was within the range of 4.26–4.76 million. The high-quality reads from individual libraries were mapped to the oyster genome; more than 20,960 mapped genes per library were determined simultaneously (Supplementary Table S1 and S2). To identify the transcriptome difference between the oysters that displayed inter-individual variations (up to 4.3-fold) in Cu body burden, the gene expression levels in the G1-H group was compared to those of the G1-L group. Similarly, the M1-H, G2-H, and M2-H groups were compared with the M1-L, G2-L, and M2-L groups, respectively. Furthermore, the DEGs in the gills of groups G1-H and G1-L intersecting with that between G2-H and G2-L (G1-L *vs.* G1-H ∩ G2-L *vs.* G2-H) were evaluated. The DEGs of the mantle in the similar treatments were assessed in relation to M1-L *vs.* M1-H ∩ M2-L *vs.* M2-H. A total of 139 DEGs between the high and low-Cu concentrations in the gills were identified (Fig. 2a). Among these genes, 34 (24.5%) were up-regulated and 105 (75.5%) were down-regulated in the gills of high-Cu concentration. In addition, 244 DEGs were detected in the mantle tissues (Fig. 2b), among which 195 (79.9%) were upregulated and 49 (20.1%) were down-regulated in the high-Cu mantles. All these genes (Supplementary Table S3) were grouped into 13 functional categories, namely, biological adhesion, biological regulation, development process, cellular process, metabolic process, immune system process, localization, response to stimulus, pigmentation, growth, multicellular organismal process, death, and cellular component organization (Fig. 2c).

### Genes related to the individual differences of Cu enrichment

AgriGO and KEGG functional enrichment analysis was used to determine the gene function of these DEGs (Supplementary Tables S4 and S5). The functional categories of these DEGs in the gills were related to lipid transport and neurotransmitter transport (selected functions in Table 1). There were 2 genes that encoded for apolipophorin or similar proteins enriched in lipid transport (Table 1), and were upregulated in the gills of high-Cu concentration. One gene encoding for the excitatory amino acid transporter 1 (EAAT 1) was specifically down-regulated in the gills of high-Cu concentration.

The functional categories of the high-Cu enrichment mantles genes were related to lipid transport, neurotransmitter transport, vesicle, response to chemical stimulus, ATPase activity, and copper ion binding (Table 1). In the lipid transport group, 2 genes encoding vitellogenin (VTG) or apolipophorin (apoLp) proteins were upregulated. In the neurotransmitter transport group, genes encoding the proline transporter and GABA transporter 2 were also identified. The gene encoding the protein, namely the ATP-binding cassette sub-family A member 3 (ABCA3), had been implicated in vesicle transport. Two genes encoding copper ion-binding proteins were specifically upregulated in the high-Cu enrichment mantles. There were four genes that were enriched in response to the chemical stimulus group, namely those encoding for the multidrug resistance protein 1 (ABCB1), multidrug resistance protein 3 (ABCB3), porphobilinogen deaminase, and UDP-glucose 6-dehydrogenase. Genes encoded in ATPase activity function included ABCB1, ABCB3 and ABCA3.

### Validation of DGE data by quantitative RT-PCR

To validate the quantitative data of the DGE libraries, we quantified the expression levels of 12 selected genes from the gills or mantle DGE libraries by qRT-PCR. The expressions of these genes measured by qRT-PCR agreed well with the Tag-seq analysis pattern (Supplementary Fig. S1).

### Inhibition of Cu enrichment by interference of *GAT* genes

**Figure 3:**
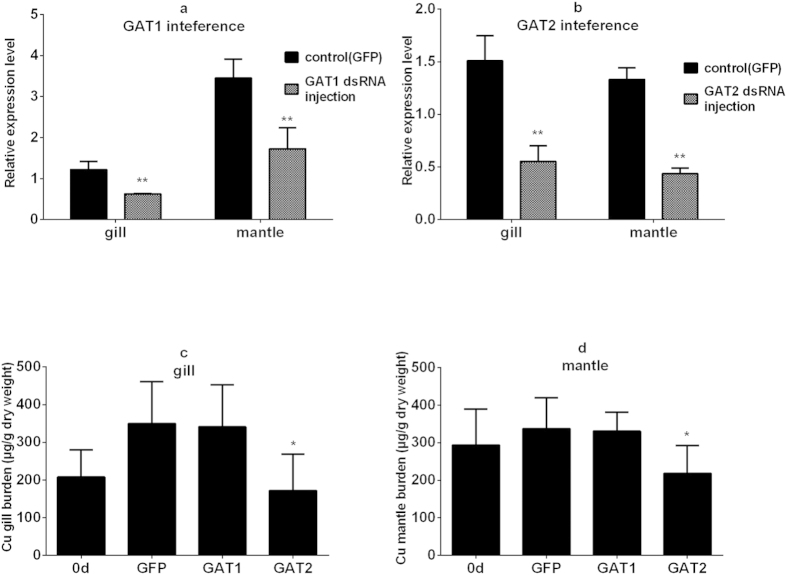
GAT1/GAT2 RNA interference. The efficiency of GAT1 (**a**) and GAT2 (**b**) RNA interference in gill and mantle following RNAi treatment. Each column represents mean ± s.d. (n=3); **p <0.01 (pair-wise t test).Cu concentration in the gills (**c**) and mantles (**d**) following GAT1/GAT2 RNA interference. Each column represents mean ± s.d. (n=8); *p <0.05 (pair-wise t test).

The function clustering entries related to the behavior, Cu iron transport and Cu stored components were the potential candidates in explaining the Cu individual variation in oysters. The neurotransmitter transport may regulate the neurotransmitters and eventually the oysters’ behavior. In the mantle, expression of *GAT2* was significantly increased in high-Cu accumulating individuals compared to the low-Cu accumulating oysters. The GABA transporters (GAT1, GAT2, GAT3, and BGT1) all potentially controlled the action of transmitter GABA in the nervous system^17^. To gain more insight into the roles of GAT2 in mediating Cu accumulation, we further conducted RNA interference experiments. Using qRT-PCR, we monitored the *GAT1* and *GAT2* transcript abundance 48 h after the first dsRNA injection. In this protocol, the receptor transcript abundance in the ds*GAT1* and ds*GAT2* injected oyster gills and mantles decreased significantly by 33–51% as compared to the control oysters (Fig. 3a,b). The gills and mantles were also collected and their Cu concentrations were analyzed after a series of four injections to further determine the roles of the *GAT1/GAT2* gene in Cu accumulation. Compared to the tissue Cu concentration before injection (0 d column), the control batch showed an increase in the gills and mantles (Fig. 3c,d), indicating that the oysters were in the process of Cu accumulation. Surprisingly, *GAT2*-silenced oysters showed a significant reduction in Cu concentration in both the gills and mantles (Fig. 3c,d), thus the silencing of the GABA transport-related *GAT2* gene may reduce the Cu accumulation.

### Functional analysis of GABA in Cu accumulation by injection of GABA and GABA receptor agonists and antagonists

**Figure 4:**
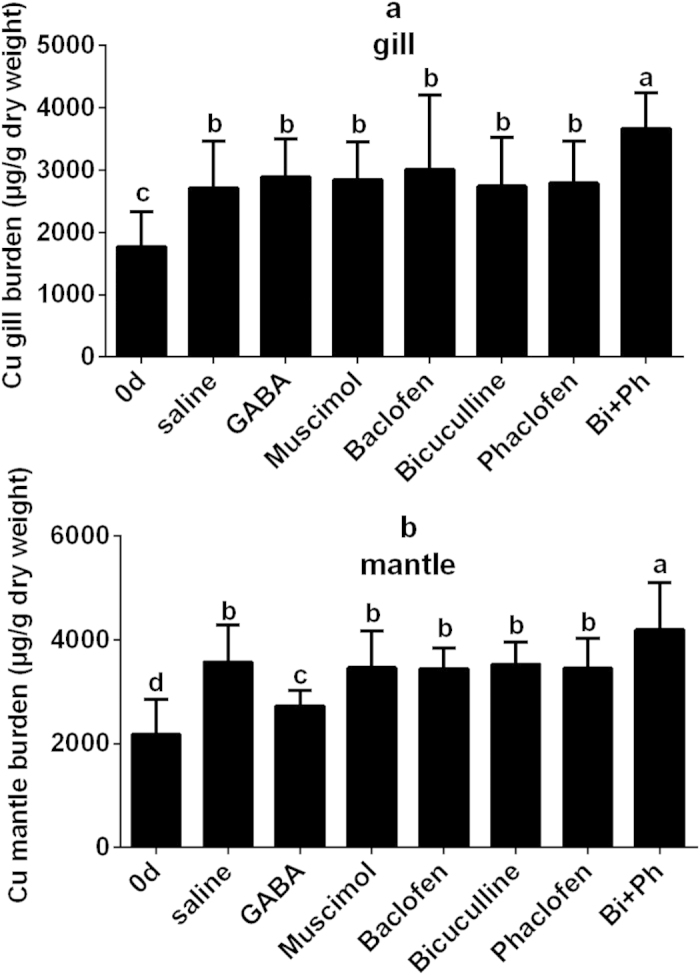
GABA, GABA receptor agonists and antagonists effect on Cu accumulation in the oysters. (**a**) Gill Cu burden after GABA, GABA receptor agonists and antagonists injection. (**b**) Mantle Cu burden after GABA, GABA receptor agonists and antagonists injection. Each column represents mean ± s.d. (n=12); p <0.05 (one-way ANOVA followed by LSD and Duncan post-hoc comparisons). Different letters represent significant difference between two treatments.

GAT2 is a promising candidate for peripheral GABA uptake and is capable of rapidly absorbing the synaptic cleft and extracellular fluid of GABA, resulting in the termination of the GABA synaptic transmission process^18^. To further confirm the effect of GAT2 on gill and mantle Cu accumulation through the regulation of GABA, we conducted another Cu accumulation study by GABA as well as GABA receptor agonist and antagonist injection. Compared to the tissue Cu concentrations before injection (0 d column), the control group displayed an increase in Cu accumulation in the gills and mantles (Fig. 4a,b). GABA, GABA receptor agonists (muscimol, baclofen), and a single GABA_A_ /GABA_B_ receptor antagonist (bicuculline or phaclofen) injection had no significant effect on Cu accumulation in the gills. However, gill Cu concentrations increased significantly after GABA_A_ and GABA_B_ receptor antagonists (bicuculline + phaclofen) treatments (Fig. 4a). These drugs imparted a similar effect on mantles, except for GABA. The Cu concentrations of the mantles were significantly reduced after GABA injection (Fig. 4b).

## Discussion

Phenotypic differences resulting from gene expression variations have been observed in various species^19^. In the present study, two individual variation groups were constructed, and the intersection of the two sets of DGE results generated from these groups were used to validate the results. Earlier studies suggested that biodynamics played important roles in Cu accumulation, inter-species and intra-species differences in Cu body burdens in oysters^5^,^10^,^11^,^20^. The present study showed that genes enriched in neurotransmitter transport functional category were differentially expressed in the mantles and gills (Table 1). Specifically, the *EAAT1* gene was down-regulated in the high-Cu gills. EAAT1 is responsible in terminating the excitatory signal by removing (or absorbing) glutamate, which is the principal excitatory neurotransmitter secreted by the neuronal synaptic cleft into neuroglia and neurons^21^. In contrast, the *GAT2* gene was up-regulated in the high-Cu mantle. GAT2 is capable of rapidly absorbing the synaptic cleft and extracellular fluid of GABA, resulting in the termination of the GABA synaptic transmission process 18,22. GABA is the major inhibitory neurotransmitter of both vertebrates and invertebrates, and may play a signaling role in peripheral organs. However, GABA has been shown to elicit both inhibitory and excitatory actions in the central neurons in mollusca^23^. To gain further insight into the roles of GAT2 in mediating Cu accumulation, we interfered both the *GAT1* gene, which was not differentially expressed, and the *GAT2* by dsRNA injection to detect the changes in oyster Cu accumulation. The oysters harboring an interfered *GAT2* gene showed a significant reduction in mantle and gill Cu concentrations as compared to the control group (Fig. 3). This finding suggested that the differences in *GAT2* transcriptional expression could lead to changes in Cu concentrations. It is noteworthy that *GAT2*-silenced oysters even showed a reduction in Cu concentration compared to the initial tissue concentration before injection (0 d column). Thus, Cu efflux was presumably higher than the Cu uptake after *GAT2* gene interference. We then further extended the Cu accumulation study by GABA, as well as GABA receptor agonist and antagonist injection (Fig. 4). Injection of a GABA_A_ receptor antagonist combined with a GABA_B_ receptor antagonist significantly facilitated the mantle and gill Cu bioaccumulation, whereas a single receptor antagonist did not produce any significant effect. These data suggested that the oysters improved their Cu bioaccumulation when GABA was suppressed. In addition, the GABA_A_ and GABA_B_ receptor were both involved in this regulatory process. GABA significantly reduced the mantle Cu concentration, whereas GABA showed no significant effect on gill and receptor agonists showed no significant effect on gill and mantle Cu accumulation. Previous studies indicated that the longest duration of behavioral change caused by GABA and receptor agonists injection in mollusca was 200 min^24^. The Cu accumulation ability in the gills was weaker than that in the mantles (Fig. 4, 0 d and saline groups). Unlike mantles, the action time of GABA may not be enough as compared to the whole Cu exposure time in gills. Therefore, there was no significant Cu burden change after GABA injection in gills. Similarly, the dose and injection frequency of the receptor agonist were not sufficient to generate a significant or persistent change in oyster behavior. It thus appeared that the differences in *GAT2* transcriptional expression regulated the GABA, resulting in changes in Cu accumulation in oysters. Such effect was probably caused by the behavioral changes observed with GABA administration.

**Figure 5:**
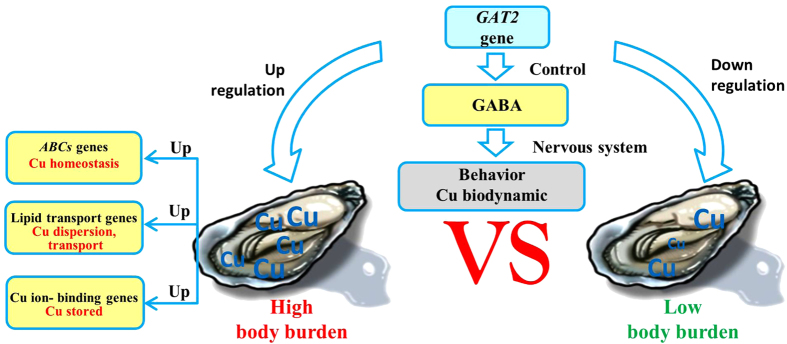
The sketch map of important functional genes related to the inter-individual variations of Cu in oysters.

GABA administration can cause a variety of behavioral changes that may be manifested by the combined effects on the neural circuitry for feeding activity, locomotion, respiration, male mating, and defensive behavior in some species of mollusks. For example, GABA induced the movements of buccal and radular muscles in *Lymnaea stagnalis*, resulting in an excitatory effect on the feeding pattern and motor activity^24^,^25^. In oysters *Crassostrea virginica*, GABA worked at both the cerebral ganglion and visceral ganglia as an inhibitory ganglionic neurotransmitter to inhibit HT neurons that innervated the gills and speed up the beating of the lateral gill cilia^26^. The feeding activity, adductor muscle, and mantle movement of oysters may also be regulated by GABA. The filtration activity is closely related to the dissolved uptake of Cu^11^, and the adductor/mantle muscles can control the extent of contact between soft tissues and seawater using shell. Therefore, when the *GAT2* gene was upregulated in some individuals, inhibitory and excitatory actions in the central neurons were elicited by GABA suppressed. We therefore inferred that neurotransmitters, especially GABA, which was regulated by GAT2, can change the oyster behavior, which may have an impact on the biodynamics of Cu (Fig. 5).

Excess Cu can induce redox reactions by generating reactive oxygen species (ROS)^27^. Most animals utilize the protective ROS-scavenging enzymes such as superoxide dismutase (SOD) and catalase (CAT) to deal with the elevated levels of ROS. The expression of proteins or genes involved in antioxidative stress (SOD, CAT, extracellular superoxide dismutase: ECSOD, cytochrome c oxidase, glutathione transferase: GST) can be stimulated by acute or chronic Cu exposure^6^,^28^. Similarly, genes encoding for heat shock proteins (HSPs), which play a vital role in the transport, folding, and assembly of proteins, are induced by various casual agents such as metals^12^,^29^,^30^. According to our data, all these genes (SOD, CAT, ECSOD, GST, and HSPs) were detected in the eight DGE libraries, but were either not or differentially expressed in very small quantity (Supplementary Table S6). Therefore, difference of oxidative stress among oysters with 4-fold difference of Cu body burdens was not obvious. It was possible that they might use transporter proteins and detoxification enzymes to remove the excessive Cu and maintain Cu homeostasis within cells^31^.

Previous studies suggested that the ATP-binding cassette transporter (ABC) superfamily was important in the maintenance of cellular metal homeostasis. ABC was composed of eight subfamilies and interacted with a wide range of chemicals including metals by pumping them across the cell membrane. These cellular activities were also called multidrug resistance^32^,^33^,^34^. ABCB1 is a drug efflux transporter of the ABCB subfamily functioning in cellular efflux and intracellular resistance to metals. Recently, ABCB1 was reported as a metal (Cu, Zn, and Cd) efflux transporter in the copepod *Tigriopus japonicus*, Asiatic clam *Corbicula fluminea*, and earthworm *Eisenia fetida*^35^,^36^,^37^. We also found that high-Cu mantles harbored these upregulated genes involved in vesicular transport, response to chemical stimulus, and ATPase activity (Table 1). The genes within these 3 groups encode for ABCB1, ABCB3, and ABCA3 all belonged to the ABC superfamily, suggesting that it played an important role in oysters by maintaining the cellular Cu homeostasis.

Metallothioneins (MTs) with thiol groups (–SH) of cysteine residues were important in metal detoxification by binding with metals in aquatic invertebrates, including bivalves^38^,^39^,^40^. However, some studies have shown that Cu imparted no effect on MTs in oysters, and MTLP played a minor role in binding Cu in oysters. Cellular debris was the main subcellular fraction for Cu binding^11^,^41^. In the present study, the genes encoding MTs were detected, but were not differentially expressed in the gills or mantles (Supplementary Table S6). Among the high-Cu mantle upregulated genes, two genes in the Cu ion-binding gene functional category were enriched (Table 1) and encoded as the protein L-ascorbate oxidase and laccase-2. Interestingly, laccases contained four Cu-binding sites and catalyzed the oxidation of a wide variety of phenolic substrates found in various plants, fungi, and microorganisms, but they were generally not found in aquatic invertebrates^42^,^43^,^44^. The upregulated expression indicated that laccase-2 may potentially bind with Cu for either detoxification or export. Consequently, MTs may not be the major detoxification in the oysters, and excessive Cu may be stored in other components such as laccase.

Our study also detected upregulated genes enriched in lipid transport functional category (Table 1). These encoded VTG and apoLp proteins have been used as biomarkers for metal exposure^45^,^46^. Cu exposure of *Paracyclopina nana* resulted in the time-dependent upregulation of VTG transcription^47^. VTG is a large precursor protein of egg yolk vitellin present in oviparous vertebrates and invertebrates^48^. In several insect species, VTGs are primarily synthesized in the fat bodies in sex-, tissue-, and stage-specific manners. After synthesis, they are secreted into the hemolymph and then sequestered by competent oocytes via receptor-mediated endocytosis^49^,^50^. VTG functions as a carrier protein for inorganic phosphates, lipids, carbohydrates, and metals, in addition to its role as a yolk precursor molecule^51^. Ghosh and Thomas reported that VTGs in estradiol-treated male red drum (*Sciaenopsocellatus*) contained calcium, magnesium, zinc, iron, and Cu, and most of the Cd had been incorporated in the ovaries, suggesting that this was an important route for metal accumulation in ovarian tissues^52^. In our study, VTGs and apoLp genes were upregulated in the gills and mantle of high-Cu oysters, and might play an important role in the transport or dispersion of excess Cu.

Our results showed that the neurotransmitter transport genes especially *GAT2* gene can regulate the inhibitory and excitatory actions elicited by GABA. The resulting behavioral changes may have an effect on Cu absorption and biodynamics, and led to individual variations in Cu accumulation. In the high-Cu oysters, ABCB1, ABCB3, and ABCA3 maintained the cellular Cu homeostasis to protect from Cu toxicity; VTGs might combine Cu with other molecules and transport or eliminate the excess Cu, while excessive Cu may be stored in components such as laccase-2 (Fig. 5). These potential genes should be further examined to confirm their functions not only in transcriptional level but also in translational level.

## Materials and Methods

### Oysters and Cu measurement

Fujian oysters, *C. angulata*, of similar body weight (15.7–24.5 g) and shell length (5.5–7.5 cm) were collected from an oyster farming site in Gangkou village (23° 42’ N, 117° 19’ E), Zhaoan Bay, Fujian Province, China. All the experimental individuals came from the same group of nine months old. The culture site was considered to be relatively free from anthropogenic Cu influences. The measured Cu concentrations in the seawater from the Zhaoan Bay were 0.39–1.37 μg/L, and in the sediments were 11.9–34.2 μg/g^53^. Sixty individuals were directly dissected and measured for Cu concentrations in the gills and mantles by inductively coupled plasma-mass spectrometry (ICP-MS), using methods described in previous study^5^,^6^. Specifically, six oysters with the highest Cu concentrations in their gills or mantles were pooled and flagged as G1-H group and M1-H, respectively. Six oysters with the lowest Cu concentrations in their gills or mantles were pooled and flagged as G1-L group and M1-L, respectively (Fig. 1).

To avoid the effects of environmental factors on the DGE results, oysters were also exposed to Cu (CuCl_2_). Before the exposure, oysters were kept in tanks containing natural seawater and acclimated for 7 days. The seawater was filtered through 0.45-μm filter (salinity: 28–30%; 26–28 °C). During the exposure, the seawater was renewed daily, and the oysters were fed commercial clean algal powders of *Chlorella* at a rate of approximately 2% of their soft tissue dry weight per day. A stock solution of 10 mg/L Cu (CuCl_2_) was prepared with Milli-Q-filtered water. Three groups of 60 individuals per tank (80 L) were exposed to 30 μg Cu/L for 30 days. At the end of exposure, 60 individuals (20 individuals per tank) were randomly selected. Similarly, the G2-L and M2-L groups were selected from 6 oysters that had the lowest Cu body concentrations, whereas G2-H and M2-H groups had the highest Cu body concentrations (Fig. 1).

### DGE library construction and sequencing

Approximately 100 mg of tissue from each of the six samples per group was collected for individual RNA extraction. Total RNA was extracted separately using Trizol reagent (Gibco BRL, USA), quantified using NanoDrop ND-2000 (Thermo Scientific, Les Ulis, France), and assessed by agarose gel electrophoresis.

Eight tag libraries from the non-exposed oysters (G1-H, G1-L, M1-H, and M1-L) and exposed oysters (G2-H, G2-L, M2-H, and M2-L) were prepared for DGE, which was performed in parallel using the Digital Gene Expression Tag Profile Kit (Illumina). The samples were prepared following the protocol described in the previous study^16^. The mRNA was enriched by using the oligo (dT) magnetic beads, further fragmented into short fragments, and mixed with the fragmentation buffer. Then, the first strand of cDNA was synthesized by using random hexamer primers based on these fragments. The second strand was synthesized using buffer, dNTPs, RNase H, and DNA polymerase I. After the double-stranded cDNA was purified with magnetic beads, end reparation and 3’-end single nucleotide A (adenine) addition was then performed. Finally, sequencing adaptors were ligated to the strands. Also, the fragments were enriched by PCR amplification. During the QC step, Agilent 2100 Bioanalyzer and ABI StepOnePlus Real-Time PCR System were used to quantify the sample library. Finally, the suitable library products were sequenced using IlluminaHiSeqTM 2000. Raw sequence data are available in the NCBI’s Gene Expression Omnibus (GEO) database under the accession number GSE65653.

### Aligning DGE tags to the reference transcriptome

To map the DGE tags, we filtered all the sequenced raw data with Perl scripts to remove low-quality tags (i.e., tags with unknown nucleotide “N”), empty tags (i.e., no tag sequence between the adaptors), and tags with only one copy number (which might have resulted from sequencing errors). *C. angulata* was considered a subspecies of *Crassostrea gigas*^54^. We also collected 80889 mRNA sequences of *C. angulata* from the NCBI and used *C. gigas* genome as database to assess the sequence identity between the two species. There were 68376 mRNA (84.5%) with more than 70% identity mapping to the database; 66522 mRNA had more than 90% identity. Such comparison suggested that the two species have a high sequence identity. For tag annotation, the clean tags containing CATG and 21-bp tag sequences were mapped to the *C. gigas* genome sequences (GEO: GSE31012) with tophat2, allowing not more than one nucleotide mismatch^55^. The clean tags were designated as unambiguous clean tags. For gene expression analysis, the number of unambiguous clean tags for each gene was calculated and normalized to the number of transcripts per million clean tags.

### DGE library characteristics and functional annotation of differentially expressed genes

Statistical analysis of the frequency of each tag in different cDNA libraries with Cufflinks was performed to compare gene expression patterns in different samples^56^. False discovery rate (FDR) was used to determine the threshold of the P value in multiple tests and analyses. A FDR of ≤0.001 and an absolute value of the log2 ratio >2 were used as the threshold to determine significant differences in gene expression. We obtained the DEGs in high Cu accumulated groups (G1-H, M1-H, G2-H, and M2-H) compared to low Cu accumulated groups (G1-L,M1-L, G2-L, and M2-L). The DEGs were used for Gene Ontology (GO) and KEGG Orthology (KO) enrichment analyses using GOstats. These GO terms were assigned to query protein sequences, which generated a broad overview of groups of genes catalogued in the three ontology vocabularies (cellular component, biological process, and molecular function). The data presented herein represented a GO analysis at level 2, illustrating general functional categories. Kyoto Encyclopedia of Genes and Genomes (KEGG) pathways were assigned to the assembled sequences using GOstats to link the online KEGG Annotation Server (http://www.genome.jp/kegg/kaas/), which provides KO assignments and pathway mapping.

### Quantitative real-time PCR (qRT-PCR)

To validate the quantitative data of DGE libraries, we quantified the expression levels of 20 selected genes from the gills or mantle DGE libraries by qRT-PCR. The total RNA samples used for the qRT-PCR assay were similar to that used in the DGE experiments. Gene-specific primers were designed according to the reference Unigene sequences using the Primer Premier 5.0 (Supplementary Table S7). The first-strand cDNA was synthesized using a PrimeScript^tm^ RT reagent Kit (TaKaRa, Dalian, China). qRT-PCR was performed according to the manufacturer’s specifications (ThermoDyNAmo Flash SYBR Green qPCR Kit, Thermo Scientific, US) on the ABI 7500 Fast Real-Time PCR System (Applied Biosystems^®^, US). The elongation factor 1α gene (*EF1α*, GenBank ID: BQ426516) was used as a normalizer (Supplementary Table S7), and the relative expression levels of genes were presented in terms of 2^-ΔΔCT^.

### dsRNA preparation and injection into oysters

According to the Pacific oyster sequence (GenBank Accession No.: EKC23164.1; EKC37107.1), GAT1 and GAT2 fragments of *C. angulata* cDNA were PCR amplified using total RNA extracted from the mantle as template. PCR fragments were sub-cloned into pMD19T-GAT1 and pMD19T-GAT2 vectors (Takara) and sequenced. pMD19T-EGFP was constructed using a 289-bp EGFP gene (EU716633.1) fragment from a pEGFP-N1 plasmid. All dsRNA synthesis assays were performed as previously described^57^.

Oysters were first acclimated in tanks containing natural seawater (salinity = 27–30%; 22–25 °C) for 7 days, afterwards 6 oysters were randomly chosen and dissected, and their gills and mantles were measured for Cu concentrations. Then the oysters were divided into 3 experiment groups (green fluorescent protein: GFP control group, GAT1, and GAT2), and were exposed to Cu at 10 μg/L for 12 days (15 individuals in 50-L tank). The shells of these oysters were drilled near the adductor muscle using a microbit (1–2 mm) to facilitate dsRNA injection. Before the experiment, the injection site was verified by injecting a similar volume of 1% methylene blue solution to determine the distribution of injected dye in the tissue^58^. After 30 min, the injected dye was distributed across the soft tissues. All the drugs were applied by means of single injection into the adductor muscle every three days, for a total of four times. The injection volume was 50 μL of saline solution with 100 μg of dsRNA. Approximately 48 h after the first injection, 3 individuals that were randomly selected from each group were dissected and used for qRT-PCR analysis to determine the effect of the injected dsRNA. The qRT-PCR protocol was the same as previously described and the *EF1α*, *β-actin* were used as normalizers. At the end of the experiment, 6 individuals were dissected and the Cu concentrations in the gills and mantles were measured. The primers used in the experiment are presented in Supplementary Table S8.

### GABA, GABA receptor agonist, and antagonist injection

Gamma-amino butyric acid (GABA), GABA_A_, and GABA_B_ receptor agonists (muscimol, baclofen), and their antagonists (bicuculline, phaclofen) were purchased from Sigma Chemical Co. (St. Louis, MO, USA). They were prepared just before use and dissolved in saline solution.

Oyster acclimation, injection protocol, and daily administration were the same as previously described. Seven experimental groups (each with 20 individuals in 80-L waters) were exposed to Cu for 12 days. All the drugs were injected into the adductor muscle of the oysters every three days, for a total of four times. The drugs and doses used in the injection for each experimental group were as follows: GABA (15 μg/g body weight), muscimol (10 μg/g body weight), baclofen (15 μg/g body weight), bicuculline (8 μg/g body weight), phaclofen (2 μg/g body weight), bicuculline (8 μg/g body weight) + phaclofen (2 μg/g body weight), and saline. These drugs and doses were used based on the preliminary experiments and earlier study^24^. At the end of the experiment, 12 individuals were dissected and the Cu concentrations in the gills and mantles were also analyzed.

## Additional Information

**How to cite this article**: Shi, B. *et al.* Transcriptome analysis of the key role of GAT2 gene in the hyper-accumulation of copper in the oyster *Crassostrea angulata*. *Sci. Rep.* **5**, 17751; doi: 10.1038/srep17751 (2015).

## Supplementary Material

Supplementary Information

Supplementary Tables

## Figures and Tables

**Table 1:** Gene ontology/pathway terms for selected genes regulated ≥two-fold with differentially expressed Cu body concentrations in the gills/mantles (low concentration *vs.* high concentration).

Gill	Expression pattern	Type^A^	G1-L Vs G1-H		G2-L Vs G2-H		Description
			FC^B^	FDR^C^	FC^B^	FDR^C^	
**Lipid transport**		P					
OYG_10014469_10040256	Upregulated		1.602509	1.13E-12	2.328729	5.9E-143	Apolipoprotein
OYG_10018340_10030734	Upregulated		1.236194	1.52E-10	1.943109	6.53E-78	hypothetical protein CGI_10018340 [Crassostreagigas]
**Neurotransmitter transport**		P					
OYG_10017841_10008053	Downregulated		−2.12145	1.17E-23	−5.3088	0	Excitatory amino acid transporter 1
**Mantle**	**Expression pattern**	**Type**^A^	**M1-L Vs M1-H**		**M1-L Vs M1-H**		**Description**
			**FC**^B^	**FDR**^C^	**FC**^B^	**FDR**^C^	
**Lipid transport**		P					
OYG_10015914_10025506	Upregulated		1.067678	0.000617	2.090331	6.7E-14	Vitellogenin/VTG
OYG_10014469_10040256	Upregulated		0.895977	1.1E-07	4.766276	2.1E-221	apolipoprotein
**Neurotransmitter transport**		P					
OYG_10000052_10033075	Upregulated		2.372763	1.16E-09	1.69651	1.48E-11	Proline transporter
OYG_10026098_10002797	Downregulated		−2.31727	2.01E-15	−1.46292	8.04E-05	Glycine transporter 2
OYG_10028335_10036542	Upregulated		1.373422	5.73E-31	1.053064	3.2E-21	GABA transporter 2/GAT2
**Vesicle**		C					
OYG_10027950_10024008	Upregulated		1.075756	7.24E-13	2.202442	4.56E-49	ATP-binding cassette sub-family A member 3/ABCA3
**Copper ion binding**		F					
OYG_10020885_10039411	Upregulated		1.499383	3.5E-32	1.701766	1.9E-09	L-ascorbate oxidase
OYG_10017371_10010587	Upregulated		1.107054	0.002036	1.43978	1.89E-11	Laccase-2
**ATPase activity**		F					
OYG_10027950_10024008	Upregulated		1.075756	7.24E-13	2.202442	4.56E-49	ATP-binding cassette sub-family A member 3/ABCA3
OYG_10000886_10006267	Upregulated		2.263479	1E-06	2.052857	1.65E-06	Multidrug resistance protein 1/ABCB1
OYG_10002457_10026801	Upregulated		1.792962	4.94E-10	1.89126	1.07E-09	Multidrug resistance protein 3/ABCB3
**Response to chemical stimulus**		P					
OYG_10000886_10006267	Upregulated		2.263479	1E-06	2.052857	1.65E-06	Multidrug resistance protein 1/ABCB1
OYG_10002457_10026801	Upregulated		1.792962	4.94E-10	1.89126	1.07E-09	Multidrug resistance protein 3/ABCB3
OYG_10022618_10024381	Upregulated		1.365183	1.95E-22	2.727761	1.19E-08	Porphobilinogen deaminase
OYG_10004131_10027936	Upregulated		1.406247	5.84E-09	1.229159	4.59E-06	UDP-glucose 6-dehydrogenase

^A^Type: P, biological process F, molecular function C, Cellular Component.

^B^FC: log2 fold change.

^C^FDR: false discovery rate.
