# Supplementary material for: Transcriptome analysis of the key role of GAT2 gene in the hyper-accumulation of copper in the oyster Crassostrea angulata
Source: Sci Rep. 2015 Dec 9;5:17751. doi: 10.1038/srep17751 (PMC4673431; doi:10.1038/srep17751)
Supplement: Supplementary Information [file srep17751-s1.doc]

**Transcriptome analysis of the key role of GAT2 gene in the hyper-accumulation of copper in oyster *Crassostrea angulata***

Bo Shi^1,2^, Zekun Huang^1,2^, Xu Xiang^3^, Miaoqin Huang^1,2^, Wen-Xiong Wang^4^, Caihuan Ke^1,2,*^

^1^State Key Laboratory of Marine Environmental Science, Xiamen University, Xiamen 361102, PR China

^2^ College of Ocean and Earth Sciences, Xiamen University, Xiamen 361102, PR China

^3^School of Life Sciences, Xiamen University, Xiamen 361102, PR China

^4^Division of Life Science, The Hong Kong University of Science and Technology, Hong Kong, PR China

^*^Address correspondence to: Caihuan Ke, College of Ocean and Earth Sciences, Xiamen University, Xiamen 361102, PR China. Tel: +86-0592-2187420; Fax: +86-0592-2880803; E-mail: [chke@xmu.edu.cn](mailto:chke@xmu.edu.cn)

Number of pages: 6

Number of figures: 1 (Figure S1)

Number of tables: 4 (Table S1, S5, S7, S8)

Table S2, S3, S4 and S6 are in the Excel file (Table S2, S3, S4, S6. xls) since the database is big.


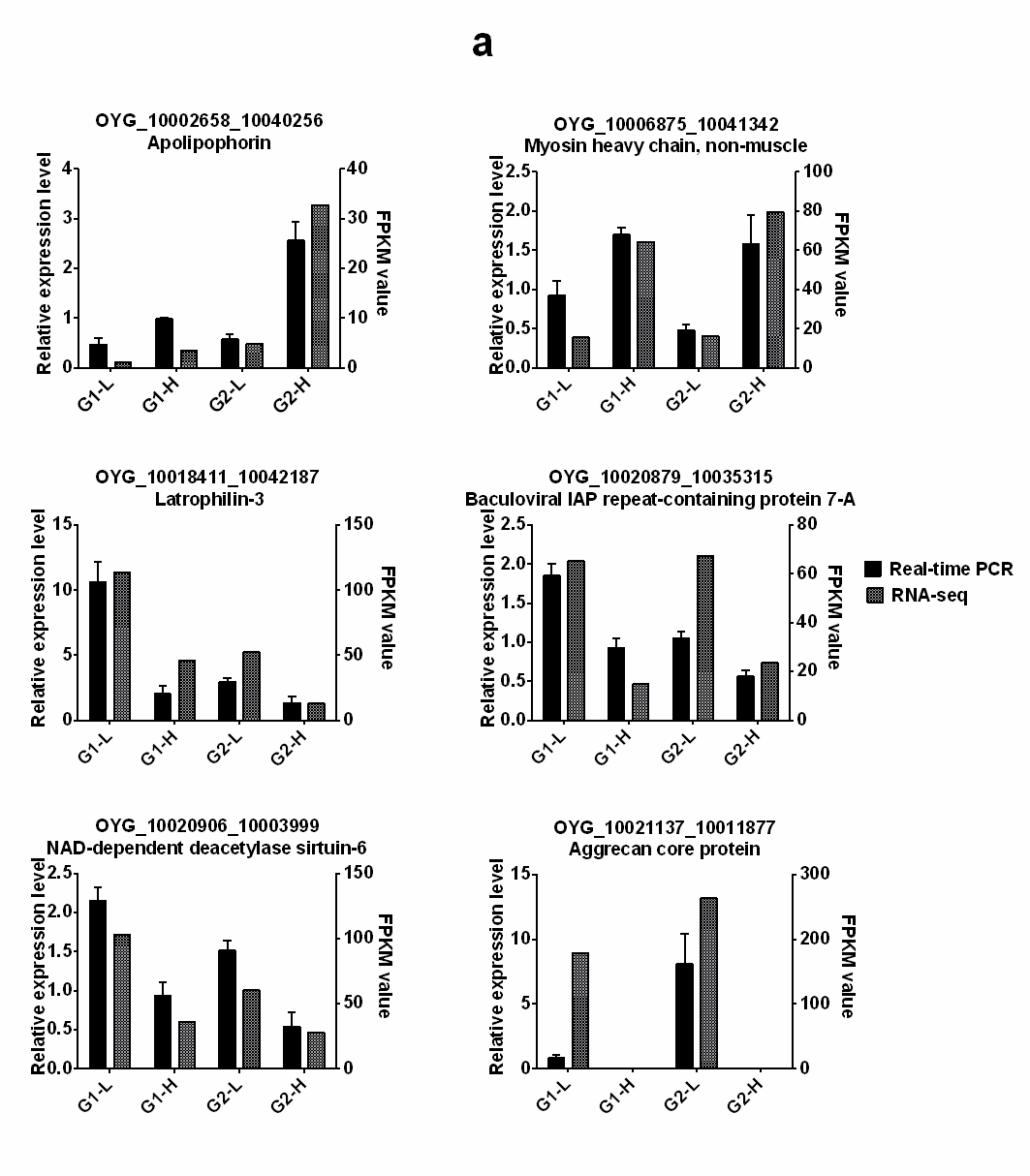

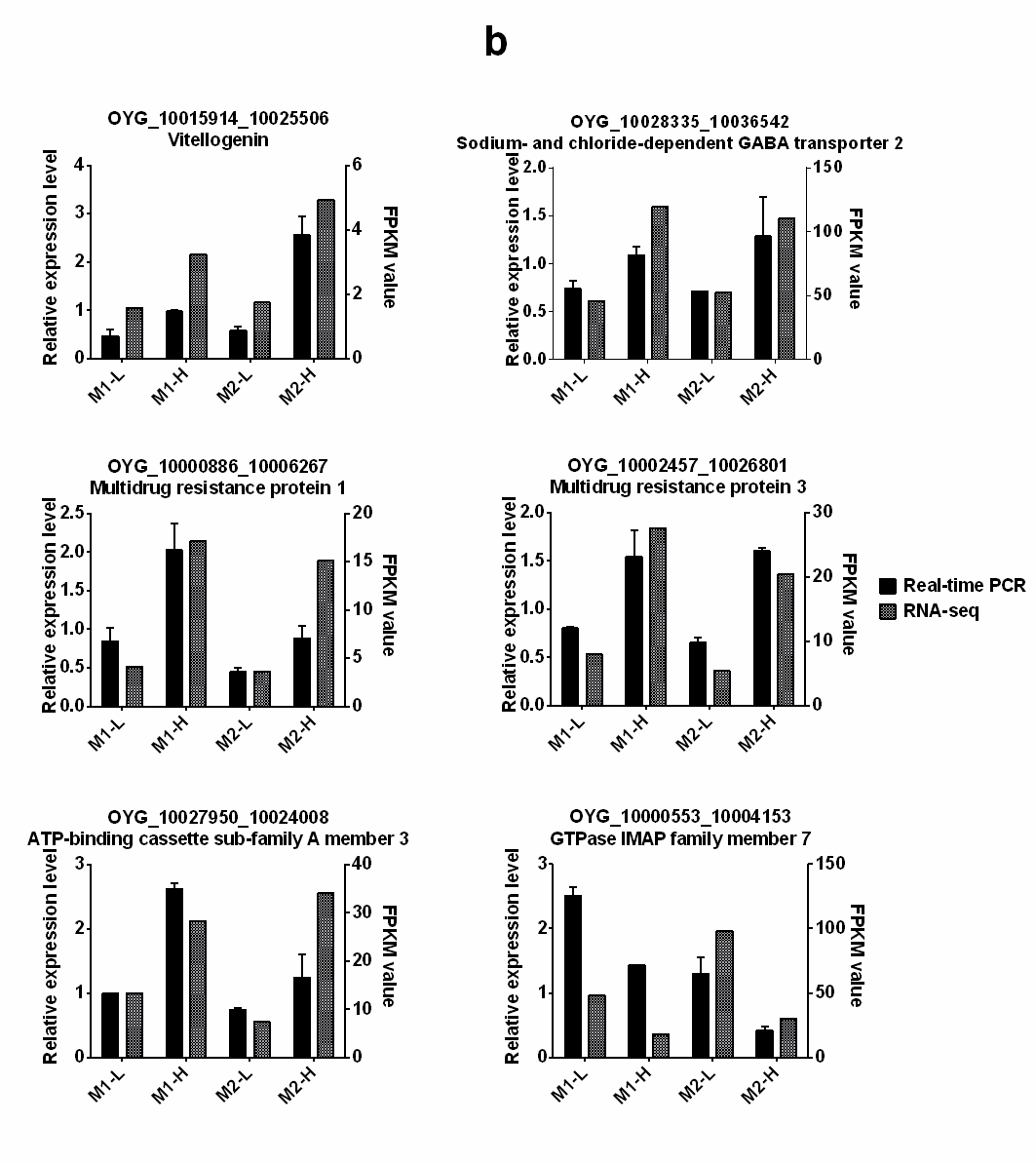


**Figure S1.** Comparison of transcription measurements by Illumina sequencing and quantitative real-time reverse transcription-PCR (qRT-PCR) assays. (a) Comparative analysis of six candidate genes expression level by qRT-PCR and RNA-seq in gill. (b) Comparative analysis of six candidate genes expression level in mantle. Error bars indicate the standard deviation. *EF1α* was used as an endogenous control.

**Table S1. Mapping results of RNA-Seq reads of gill and mantle samples**

| **Samples** | **Total number of filtered single-end reads** | **Percentage of mapped reads (%)** | **Percentage of unique mapped reads (%)** | **Total number of mapped genes** |
| --- | --- | --- | --- | --- |
| G1-L | 6,217,720 | 4,635,512 (74.55%) | 3,259,886 (52.43%) | 21,103 |
| G1-H | 6,315,216 | 4,649,750 (73.63%) | 3,411,370 (54.02%) | 21,257 |
| G2-L | 5,853,876 | 4,282,870 (73.16%) | 3,350,228 (57.23%) | 21,318 |
| G2-H | 5,928,195 | 4,256,134 (71.79%) | 3,388,105 (57.15%) | 20,960 |
| M1-L | 6,171,404 | 4,720,488 (76.49%) | 3,168,773 (51.35%) | 21,410 |
| M1-H | 6,061,038 | 4,575,894 (75.50%) | 3,251,727 (53.65%) | 21,435 |
| M2-L | 6,267,988 | 4,764,793 (76.02%) | 3,338,322 (53.26%) | 21,164 |
| M2-H | 6,004,064 | 4,457,049 (74.23%) | 3,378,489 (56.27%) | 21,508 |

| Table S2, S3, S4 and S6 are in the Excel file (Table S2,S3,S4,S6.xls) since the database is big.  **Table S5.** KO enrichment analysis of DEGs of higher Cu concentration compared with lower Cu concentration in gills and mantles. | | | | | | |
| --- | --- | --- | --- | --- | --- | --- |
| **KEGGID** | **Term** | | **P value** | **Odds Ratio** | **Number in input list** | **Number in Ref** |
| 5200 | | Pathways in cancer | 0 | 3.157 | 19 | 390 |
| 4064 | | NA | 0 | 4.503 | 10 | 142 |
| 4210 | | Apoptosis | 0 | 4.435 | 10 | 144 |
| 5222 | | Small cell lung cancer | 0 | 3.856 | 11 | 181 |
| 5145 | | Toxoplasmosis | 0.001 | 3.041 | 12 | 247 |
| 4510 | | Focal adhesion | 0.002 | 2.221 | 21 | 598 |
| 5166 | | NA | 0.005 | 2.663 | 11 | 255 |
| 4724 | | NA | 0.006 | 3.49 | 7 | 124 |
| 4977 | | Vitamin digestion and absorption | 0.006 | 4.671 | 5 | 67 |
| 4120 | | Ubiquitin mediated proteolysis | 0.007 | 2.535 | 11 | 267 |
| 5206 | | NA | 0.008 | 2.218 | 14 | 389 |
| 4975 | | Fat digestion and absorption | 0.009 | 4.256 | 5 | 73 |
| 4360 | | Axon guidance | 0.01 | 2.885 | 8 | 170 |
| 4011 | | MAPK signaling pathway - yeast | 0.024 | 5.213 | 3 | 36 |
| 5144 | | Malaria | 0.026 | 3.172 | 5 | 96 |
| 500 | | Starch and sucrose metabolism | 0.028 | 3.712 | 4 | 66 |
| 906 | | Carotenoid biosynthesis | 0.035 | 56.804 | 1 | 2 |
| 521 | | Streptomycin biosynthesis | 0.042 | 6.714 | 2 | 19 |
| 2010 | | ABC transporters | 0.047 | 3.106 | 4 | 78 |
| 4330 | | Notch signaling pathway | 0.048 | 2.39 | 6 | 151 |

| **Table S7.** Information of primers used in qRT-PCR analysis | | |  |
| --- | --- | --- | --- |
| **Gene ID** | **GENE Description** | **Forward Primer (5'-3')** | **Reverse Primer (5'-3')** |
| BQ426516 | EF1α | ACCACCCTGGTGAGATCAAG | ACGACGATCGCATTTCTCTT |
| OYG_10002658_10040256 | Apolipophorin | CAACGGTGCCAATGAAGAG | TGGAGGTAAGCAGCTAAACG |
| OYG_10006875_10041342 | Myosin heavy chain | AGAAGAAACCAATAAAAGAGTG | GATGTTGTAAAGTACAGAGGCG |
| OYG_10018411_10042187 | Latrophilin-3 | GGGCTTTATGTTTTCTCTTGG | TCACTTTATCTCCTGTGTTTA |
| OYG_10020879_10035315 | Baculoviral IAP repeat-containing protein 7-A | GCTGAGAGAAAGAACCCCCT | GCGATGTTTGATCCAGACAT |
| OYG_10020906_10003999 | NAD-dependent deacetylase sirtuin-6 | GCTCTACGCCCAACTTACAC | CACTTCTCGCATTTCTCCAC |
| OYG_10021137_10011877 | Aggrecan core protein | AGATGTTATGTCGCACGCAG | AGAAGGGAACCTATAGTCGC |
| OYG_10015914_10025506 | Vitellogenin (VTG) | GCAGCGACAACAACCAGAA | GCGGCATAACTTCCCTAATC |
| OYG_10028335_10036542 | Sodium- and chloride-dependent GABA transporter 2 | GACCGTTCTCTCGCCTATTTT | CAGTCCTTGTGGGGTTATCAA |
| OYG_10000886_10006267 | Multidrug resistance protein 1 (ABCB1) | TTGGAGCGTTTTCTTTGGGT | CTGGACATTCTCTCTGGCGG |
| OYG_10002457_10026801 | Multidrug resistance protein 3 (ABCB3) | GCAAGACAGCCACGGAATC | CAGCATAGGCGAAGAAGAG |
| OYG_10027950_10024008 | ATP-binding cassette sub-family A member 3 (ABCA3) | AAGTTGATGGGGATGAGTGAA | AAGAAGACCAGGATGAGGGAG |
| OYG_10000553_10004153 | GTPase IMAP family member 7 | TACCAAAGCCAAAATGCCT | TTTCTCTGACCCTCTCCCA |

| **Table S8.** Sequences of primers used for cloning and qRT-PCR in RNAi experiment. | |
| --- | --- |
| **Primer name** | **Sequence (5'-3')** |
| EGFPF166 | CAGTGCTTCAGCCGCTACCC |
| EGFPR454 | AGTTCACCTTGATGCCGTTCTT |
| EGFP-T7F | TAATACGACTCACTATAGGCAGTGCTTCAGCCGCTACCC |
| EGFP-T7R | TAATACGACTCACTATAGGAGTTCACCTTGATGCCGTTCTT |
| EF1α-F | ACCACCCTGGTGAGATCAAG |
| EF1α-R | ACGACGATCGCATTTCTCTT |
| βactin-F | CTCTTTCACCACCACAGCC |
| βactin-R | GTACCTCCAGACAGGACAATA |
| GAT1F | GTCCATGCATTCTTCCCCAA |
| GAT1R | GAAAAACAGCACTCCCCACA |
| GAT1-T7F | TAATACGACTCACTATAGGGTCCATGCATTCTTCCCCAA |
| GAT1-T7R | TAATACGACTCACTATAGGGAAAAACAGCACTCCCCACA |
| GAT1-qF | TTCTTTATGTTGATTGCGGT |
| GAT1-qR | TGATACACTCTGAGAGGGCC |
| GAT2F | AGGGGTGGACATCAAGGACG |
| GAT2R | GAAAATAGGCGAGAGAACGG |
| GAT2-T7F | TAATACGACTCACTATAGGAGGGGTGGACATCAAGGACG |
| GAT2-T7R | TAATACGACTCACTATAGGGAAAATAGGCGAGAGAACGG |
| GAT2-qF | GACCGTTCTCTCGCCTATTTT |
| GAT2-qR | CAGTCCTTGTGGGGTTATCAA |
